# Calcineurin B in CD4^+^ T Cells Prevents Autoimmune Colitis by Negatively Regulating the JAK/STAT Pathway

**DOI:** 10.3389/fimmu.2018.00261

**Published:** 2018-02-19

**Authors:** Andrea Mencarelli, Maurizio Vacca, Hanif Javanmard Khameneh, Enzo Acerbi, Alicia Tay, Francesca Zolezzi, Michael Poidinger, Alessandra Mortellaro

**Affiliations:** ^1^Singapore Immunology Network (SIgN), Agency for Science, Technology and Research (A*STAR), Singapore, Singapore; ^2^Department of Microbiology and Immunology, Yong Loo Lin School of Medicine, National University of Singapore, Singapore, Singapore

**Keywords:** calcineurin B, nuclear factor of activated T cell, colitis, CD4^+^ T cells, inflammatory bowel disease

## Abstract

Calcineurin (Cn) is a protein phosphatase that regulates the activation of the nuclear factor of activated T-cells (NFAT) family of transcription factors, which are key regulators of T-cell development and function. Here, we generated a conditional Cnb1 mouse model in which Cnb1 was specifically deleted in CD4^+^ T cells (Cnb1^CD4^ mice) to delineate the role of the Cn–NFAT pathway in immune homeostasis of the intestine. The Cnb1^CD4^ mice developed severe, spontaneous colitis characterized at the molecular level by an increased T helper-1-cell response but an unaltered regulatory T-cell compartment. Antibiotic treatment ameliorated the intestinal inflammation observed in Cnb1^CD4^ mice, suggesting that the microbiota contributes to the onset of colitis. CD4^+^ T cells isolated from Cnb1^CD4^ mice produced high levels of IFNγ due to increased activation of the JAK2/STAT4 pathway induced by IL-12. Our data highlight that Cn signaling in CD4^+^ T cells is critical for intestinal immune homeostasis in part by inhibiting IL-12 responsiveness of CD4^+^ T cells.

## Introduction

Calcineurin (Cn) is a Ca^2+^-activated serine/threonine phosphatase that contains a catalytic (CnA) and regulatory (CnB) subunit. Cn is involved in many biological processes, including immune responses (1). Ca^2+^–calmodulin–Cn signaling activates the nuclear factor of activated T-cells (NFAT) family of transcription factors. Phosphorylated and inactive NFAT proteins are found in the cytosol of resting cells; once activated *via* receptor-coupled Ca^2+^ signaling, they are rapidly dephosphorylated by Cn, translocate to the nucleus, and activate gene transcription.

The Ca^2+^–Cn–NFAT-signaling pathway is critical in regulating several T-cell functions, including initiating the expression of cytokines, chemokines and their receptors, and master regulators for T helper (Th)-cell differentiation (2). NFAT in T cells also regulates a transcriptional program that induces regulatory T-cell (Treg) development and T-cell tolerance (anergy) (3–6). NFAT directs these two opposing programs by cooperating with other transcription factors that help improve its DNA-binding capacity and transcription efficiency. T-cell activation *via* T-cell receptor (TCR) and costimulatory receptor binding induces the formation of the NFAT:AP-1 enhancer complex, which regulates a large set of genes expressed in the activated T cells. Conversely, the activation of Ca^2+^ signaling alone leads to NFAT-mediated transcription of anergy-associated genes, including the ubiquitin ligases Itch, Cbl-b, GRAIL, and Tsg101 (7). Thus, the ability of the Ca^2+^–Cn–NFAT pathway to interpret and regulate both stimulatory and inhibitory signals in T cells suggests that the outcome of the immune response depends on the cell type and signaling context in which the pathway is activated.

The intestinal mucosa is a major site for dynamic interactions between the host mucosal immune system and commensal microbiota. Here, intestinal homeostasis is achieved *via* a series of control mechanisms, including the differentiation of T cells into subsets of effector and regulatory cells (8). Disrupted homeostasis is a hallmark of inflammatory bowel disease (IBD)—an immune-mediated disorder of the gastrointestinal tract, characterized by uncontrolled inflammation due to persistent, aberrant activation of the mucosal immune system (8). Ulcerative colitis (UC) and Crohn’s disease (CD) are two common forms of IBD caused by excessive effector T-cell activation that is accompanied, in some circumstances, by the altered regulation of T-cell-mediated tolerance, including defective Treg-cell activity (9).

Corticosteroids are the first-line therapy for active IBD (10), but patients with IBD refractory to steroid therapy require treatment with the Cn inhibitors cyclosporine A (CsA) or FK506 (11). Inhibiting Cn is currently the only effective therapeutic strategy to suppress memory CD4^+^ and CD8^+^ T-cell activation. Thus, Cn inhibitors are commonly used as immunosuppressants in steroid-resistant IBD, as well as anti-rejection drugs in solid-organ transplantation (12, 13). Cn inhibitors can induce rapid remission in patients with severe UC, but their efficacy in active CD is limited (14). Cn inhibitors cause unbalanced Th-cell alloreactivity and effector function (15–17), and the suppression of T-cell tolerance by reducing the Treg-cell pool, which can lead to insensitivity of T-cell subpopulations and the activation of intestinal T cells (15, 17, 18). Since there are evidences that Cn inhibitors can also modulate several other cellular processes, including protein degradation and transcriptional activity of different transcription factors, the consequences of the constitutive Cn depletion in CD4^+^ T cells in a context of intestinal inflammation remain to be determined. Moreover, CsA and FK506 elicit notable adverse effects, including permanent nephrotoxicity, pneumonia, and anaphylaxis. As such, their long-term efficacy remains to be determined (19).

Despite advances in understanding the molecular basis of the Ca^2+^–Cn–NFAT pathway in CD4^+^ T cells, most insights have been obtained from mouse models with the global deletion of NFAT1, NFAT2, NFAT4 (20), CnB (21) or CnA (22), or from mice treated with CsA or FK506 (23). Few studies have generated conditional mouse models with either NFAT or Cn deletion in only thymocytes or mature CD4^+^ T cells. Neilson and colleagues showed that Cnb1 deletion in thymocytes results in impaired positive selection affecting thymocyte development (21). The deletion of NFAT2 in CD4^+^ T cells caused a reduction of CD4^+^CXCR5^+^Foxp3^+^ follicular Tregs, leading to an augmented germinal center reaction and the onset of lupus-like disease upon immunization (24). Moreover, it was also reported that NFAT transcription factors in CD4^+^ T cells also regulate the differentiation of inducible Treg (iTreg) cells *via* the induction of Foxp3 expression, but NFAT seems to be dispensable for iTreg-cell-mediated suppressor function (25). However, none of these studies have addressed the role of Cn in the mouse intestine. Our understanding as to how the Ca^2+^–Cn–NFAT axis in CD4^+^ T cells regulates intestinal immune homeostasis and the balance between T-cell activation and tolerance is, therefore, incomplete. Considering that Cn inhibitors have broad utility in the clinic, it is important to determine the immunological consequences of persistent Cn inhibition in T cells.

Here, we generated a mouse line carrying a Cnb1 deletion in CD4^+^ T cells (Cnb1^CD4^ mice) to investigate the role of the Ca^2+^–Cn–NFAT pathway in intestinal immune homeostasis. These mutant mice exhibited spontaneous intestinal inflammation caused by an exacerbated type-1 Th (Th1)-cell effector phenotype, while Treg-cell composition and activity did not change significantly. This excessive Th1-cell response was triggered by the activity of the intestinal microbiota and could be reversed by antibiotic treatment. Gene expression microarray analysis found that the absence of Cnb1 in CD4^+^ T cells leads to an increased activation of the JAK/STAT-signaling pathway. These data indicate that the Ca^2+^–Cn–NFAT pathway contributes to intestinal immune homeostasis by negatively regulating the JAK/STAT4 pathway in CD4^+^ T cells.

## Materials and Methods

### Mice

C57BL/6-Ppp3r1^tm1Stl^/J (Cnb1^fl/fl^) mice were crossed with Tg(Cd4-cre) 1Cwi/BfluJ transgenic mice (The Jackson Laboratory; Stocks #6581 and #11336, respectively) to generate Cnb1^CD4^ mice. All experiments used congenic littermate control mice, and all mice were maintained under specific pathogen-free conditions. All experimental procedures were approved by the Institutional Animal Care and Use Committee (IACUC) of the Biological Resource Center (BRC) (A*STAR) in compliance with their Guidelines for Animal Experiments.

### Antibiotic Treatment

Full-spectrum antibiotic treatment (1 g/l ampicillin, 1 g/l streptomycin, 1 g/l vancomycin, and 0.5 g/l metronidazole; Sigma) was provided *ad libitum* in drinking water to 10-week-old mice for 4–5 weeks. The antibiotic solution was refreshed every 4 days.

### Inflammatory Histological Score

Sections of the medial colon were fixed in buffered formalin, cut to 5 µm, stained with hematoxylin and eosin, and scored in a blinded fashion. The histologic-scoring system evaluating the degree of inflammation was graded semi-quantitatively from 0 to 4 as follows: 0, no signs of inflammation; 1, very low level of leukocyte infiltration; 2, low level of leukocyte infiltration; 3, high level of leukocyte infiltration, high vascular density, and thickening of the colon wall; and 4, transmural infiltration, loss of goblet cells, high vascular density, and thickening of the colon wall.

### *In Vitro* T-Cell Polarization

Purified naïve CD45RB^high^CD62L^+^CD44^neg^ CD4^+^ T cells from the spleens of Cnb1^fl/fl^ and Cnb1^CD4^ mice were cultured under conditions to promote Th0 [5 µg/ml anti-CD3ε (clone 2C11), 1 µg/ml anti-CD28 (clone 37.51), and 20 ng/ml IL-2], Th1 [5 µg/ml anti-CD3ε, 1 µg/ml anti-CD28, 20 ng/ml IL-2, 20 ng/ml IL-12, and 10 µg/ml anti-IL-4 (clone 11B11)], Th17 [5 µg/ml anti-CD3ε, 1 µg/ml anti-CD28, 20 ng/ml IL-2, 2 ng/ml TGFβ1, 100 ng/ml IL-6, 10 µg/ml anti-IL-4, 10 µg/ml anti-IFNγ (clone R4-6A2), 65 ng/ml IL-21, 20 ng/ml IL-23, and 20 ng/ml IL-1β], type-2 Th (Th2) cell (5 µg/ml anti-CD3ε, 1 µg/ml anti-CD28, 20 ng/ml IL-2, 10 µg/ml anti-IL-12, 10 µg/ml anti-IFNγ, and 100 ng/ml recombinant IL-4), and Treg (5 µg/ml anti-CD3ε, 1 µg/ml anti-CD28, 200 U/ml IL-2, 5 ng/ml TGFβ, 2.5 µg/ml anti-IFNγ, and 2.5 µg/ml anti-IL-4) cells. Recombinant IL-1β, IL-2, IL-4, IL-6, IL-12, and IL-21 are from Peprotech, recombinant TGFβ1 and IL-23 are from R&D Systems, and all neutralizing antibodies from eBioscience. T cells were collected after 5 days and stimulated with 50 ng/ml PMA and 1 µg/ml ionomycin for 5 h, with 5 µg/ml Brefeldin A (Sigma) added for the last 4 h. Cells were stained for intracellular IFNγ, IL-17, IL-4, and Foxp3, and the proportions of CD4^+^ T cells producing these cytokines were assessed by flow cytometry.

### *In Vitro* Treg-Cell Suppression Assay

Sorted splenic-naïve CD4^+^ T cells (CD45RB^high^CD44^low^CD62L^+^) from Cnb1^fl/fl^ mice were stained with CellTrace™ Violet (Molecular Probes) and cocultured with sorted splenic dendritic cells (CD11c^high^MHCII^+^) at a 5:1 ratio in the presence or absence of sorted Treg cells (CD45RB^low^CD25^+^GiTR^+^) from Cnb1^fl/fl^ or Cnb1^CD4^ mice and plate-bound anti-CD3ε (clone 2C11, 2 µg/ml, eBioscience) and anti-CD28 (clone 37.51, 2 µg/ml, eBioscience) antibodies, and IL-2 (200 U/ml). After 4 days, proliferation of the naïve CD4^+^ T cells was measured by dye dilution. Data were analyzed using FlowJo (TreeStar Inc.).

### Isolation of Lamina Propria (LP) Leukocytes from Colon

Colons were cut longitudinally and divided into 0.5–1.0-cm segments. The segments were incubated in RPMI supplemented with 2% fetal bovine serum (FBS) for 20 min at 37^°o^C with constant stirring. After incubation, the cell suspension was passed through a 70-µM sterile strainer, and the pieces of colon tissue were transferred to a 50-ml tube containing 15-ml serum-free RPMI, shook vigorously for 30 s, and then strained twice through a 70-µM cell strainer. The remaining filtered colon fragments were washed in calcium-free and magnesium-free HBSS and treated with 1 mM EDTA in PBS for two, 20-min incubations to remove the epithelium. The tissue was then digested with 0.8 mg/ml type IV collagenase (Sigma), and leukocytes were further enriched on a 40:75% Percoll gradient (Pharmacia, Uppsala, Sweden). The interface containing the leukocytes was collected after centrifugation at 700 × *g* for 20 min.

### Cytokine Production from CD4^+^ T Cells

Mononuclear cells isolated from colonic-LP were stimulated with PMA (1 µg/ml, Sigma) and ionomycin (1 µg/ml) for 6.5 h *in vitro* in the presence of Brefeldin A (10 µg/ml, Sigma) for the final 5 h. The cells were then harvested and surface-labeled with anti-CD4 antibody before fixation/permeabilization using BD Cytofix/Cytoperm™ kits (BD Bioscience). Intracellular staining was performed for 30 min at 4^°^C using anti-IL-2 (JES5H4), IL-17 (TC11-18H10.1), IL-10 (JES5-16E3), IL-4 (BVD6-24G2), and TGFβ1 (TW7-16B4) antibodies, all from Biolegend.

For apoptosis experiments with sorted cells, DAPI^−^CD45^+^CD4^+^ T cells from colonic LP were sorted and seeded into 96-well plates for stimulation with plate-bound anti-CD3ε (clone 2C11, 5 µg/ml, eBioscience) and soluble anti-CD28 (clone 37.51, 2 µg/ml, eBioscience) antibodies in complete RPMI medium and incubated for 24 h at 37^°^C. After incubation, cell death was quantified by Annexin V and propidium iodide staining (BD Bioscience).

### Cytokines Production from Myeloid Cells

Sorted CD11c^+^MHCII^high^ positive and negative for CD11b cells were seeded into 96-well culture plates (150,000 cells/100 μl/well) in RPMI medium containing 10% FBS, 100 IU/ml penicillin, 100 µg/ml streptomycin, 2 mM glutamine, 10 mM HEPES, and 50 µM β-mercaptoethanol (all from GIBCO) and then stimulated with or without LPS from *Escherichia coli* (10 µg/ml, Invivogen) for 16–18 h. Cytokine levels were assessed by ELISA. Values indicate the mean ± standard error of two experiments (*n* = 4–6 mice/experiment).

### *In Vitro* CD4^+^CD44^low^ T-Cell Cytokine Production and Intracellular STAT4 Staining

Colonic-LP CD45^+^CD3^+^CD4^+^CD44^low^ cells were sorted and seeded into 96-well plates for stimulation with plate-bound anti-CD3ε (clone 2C11, 5 µg/ml, eBioscience) and soluble anti-CD28 (clone 37.51, 2 µg/ml, eBioscience) antibodies alone or in combination with recombinant IL-12 (100 ng/ml, Peprotech) in complete RPMI medium and were incubated for 8 h at 37^°^C. After incubation, supernatants were collected for cytokine ELISA detection.

For STAT4 staining, CD4^+^ T cells were incubated at 37^°^C for 5 h in complete RPMI medium alone or with recombinant IL-12 (100 ng/ml, Peprotech) and then fixed (4% PFA) for 30 min at room temperature. The cells were then rinsed three times in PBS for 5 min and incubated in a blocking buffer (0.1% Saponin in 1% BSA–PBS) for 30 min at room temperature. The cells were then incubated for 2 h at room temperature with purified anti-STAT4 (15A1B41, Biolegend) and IgG2b isotype (RTK4530, Biolegend) antibodies diluted in a blocking buffer, followed by two washes in a blocking buffer for 5 min and incubated overnight at 4^°^C. The following day, the cells were incubated for 1 h with a PE-conjugated secondary antibody (Thermo Scientific), then washed and mounted with Prolong gold DAPI antifade reagent (Molecular Probes).

### Statistical Analysis

Data are expressed as the means ± standard error. Two-tailed, unpaired Student’s *t*-tests or ANOVA followed by Tukey’s Multiple Comparison Test were used to compare two groups of data, as indicated. A *P* < 0.05 was considered to be statistically significant. GraphPad Prism version 5.0 was used to prepare the graphics and perform all statistical analyses (GraphPad Software, San Diego, CA, USA).

## Results

### Cn in CD4^+^ T Cells Is Required for Thymic Development and Peripheral Homeostasis of T Cells

**Figure 1:**
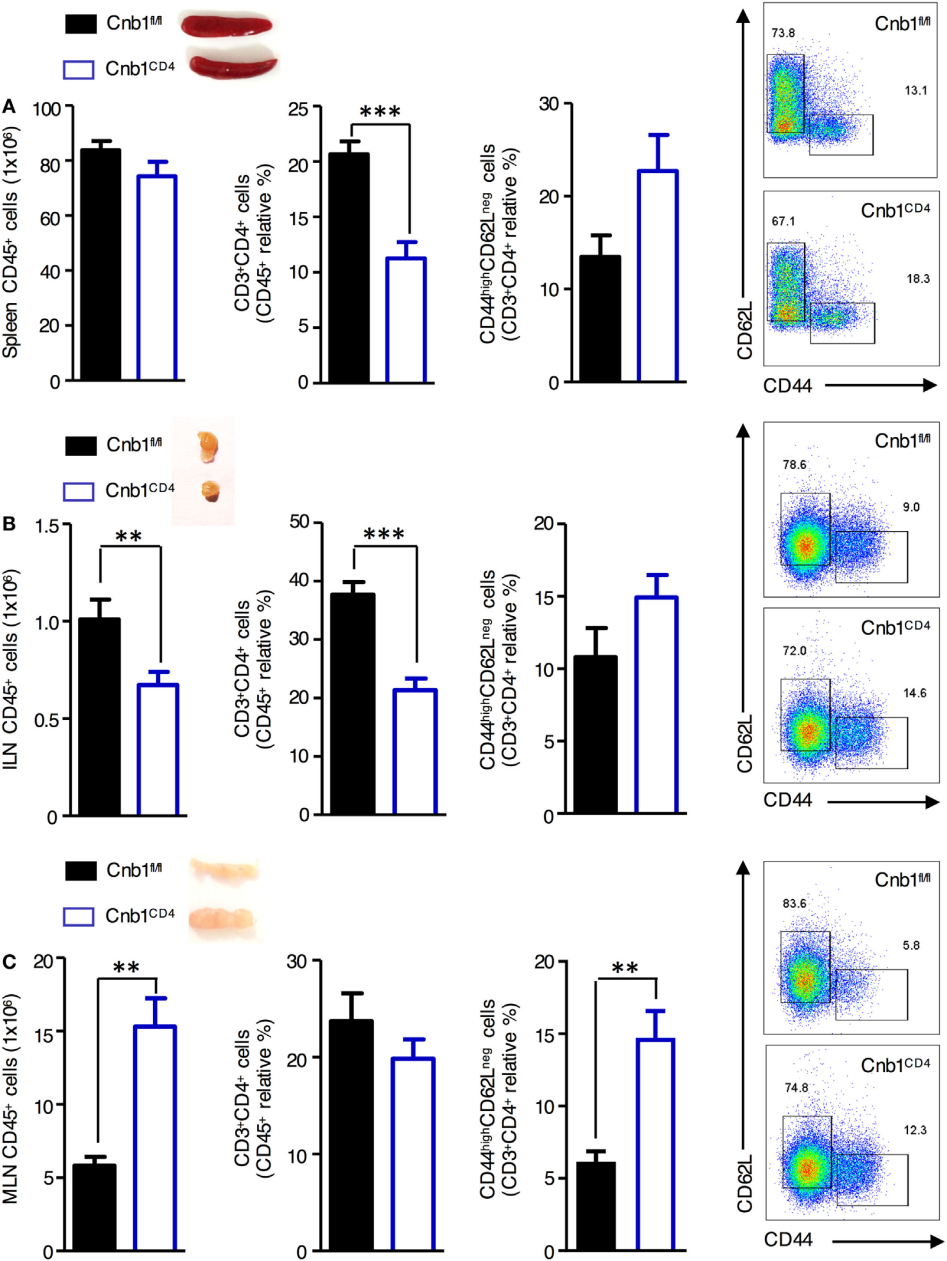
Characterization of CD4^+^ T cells from the spleen, inguinal lymph node (ILN), and mesenteric lymph node (MLN) of Cnb1^CD4^ mice. Cells were harvested from **(A)** the spleen, **(B)** ILN, and **(C)** MLN of Cnb1^CD4^ and Cnb1^fl/fl^ mice aged 5–6 weeks. Florescence-activated cell-sorting analysis was performed to characterize the total number of CD45^+^ leukocytes and antigen-experienced CD44^high^CD62L^neg^ CD4^+^ T cells. Representative dot plots of the proportions of CD44-expressing versus CD62L-expressing cells in the CD4^+^ T-cell population of Cnb1^CD4^ and Cnb1^fl/fl^ mice are shown. Data represent the means ± standard error of two independent experiments (*n* = 2–3 mice per group, per experiment). *P < 0.05; **P < 0.01; ***P < 0.005 (two-tailed, unpaired Student’s *t*-test).

To determine the importance of Cn in CD4^+^ T cells *in vivo*, we generated mice with loxP-flanked Cnb1 alleles that also express Cre recombinase under the control of the CD4 promoter (Cnb1^CD4^ mice) to delete Cnb1 in CD4^+^ T-cell populations. A reduction in CD4^+^ T-cell number in the spleen and inguinal lymph node (ILN) of 4–5-week-old Cnb1^CD4^ mice was found compared to that of control Cnb1^fl/fl^ mice (Figures 1A,B). The mesenteric lymph nodes (MLNs) of Cnb1^CD4^ mice were enlarged but the frequency of CD4^+^ T cells was normal (Figure 1C). CD4^+^ T cells obtained from the MLN of Cnb1^CD4^ mice expressed significantly higher levels of CD44 together with lower levels of the naïve T-cell marker CD62L compared to those of Cnb1^fl/fl^ mice (Figure 1C). CD8^+^ T cells were also significantly reduced (two- to threefold) in the spleen, ILN, and MLN, but these cells expressed normal levels of CD44 (Figure S1A in Supplementary Material).

Reduced CD4^+^ and CD8^+^ T-cell numbers may indicate a developmental defect of Cnb1-deficient thymocytes, as previously reported in total null mice for Cnb1 and CnAβ mice (22). Flow-cytometric analysis revealed a significant accumulation of CD4^+^CD8^+^ double-positive thymocytes with a significant decrease in the proportion of CD4^+^ and CD8^+^ single-positive thymocytes in Cnb1^CD4^ thymi compared to thymi from control mice (Figure S1B in Supplementary Material), confirming the importance of Cnb1 in T-cell development and homeostasis.

### Cnb1 Depletion in CD4^+^ T Cells Induces Spontaneous Chronic Colitis

**Figure 2:**
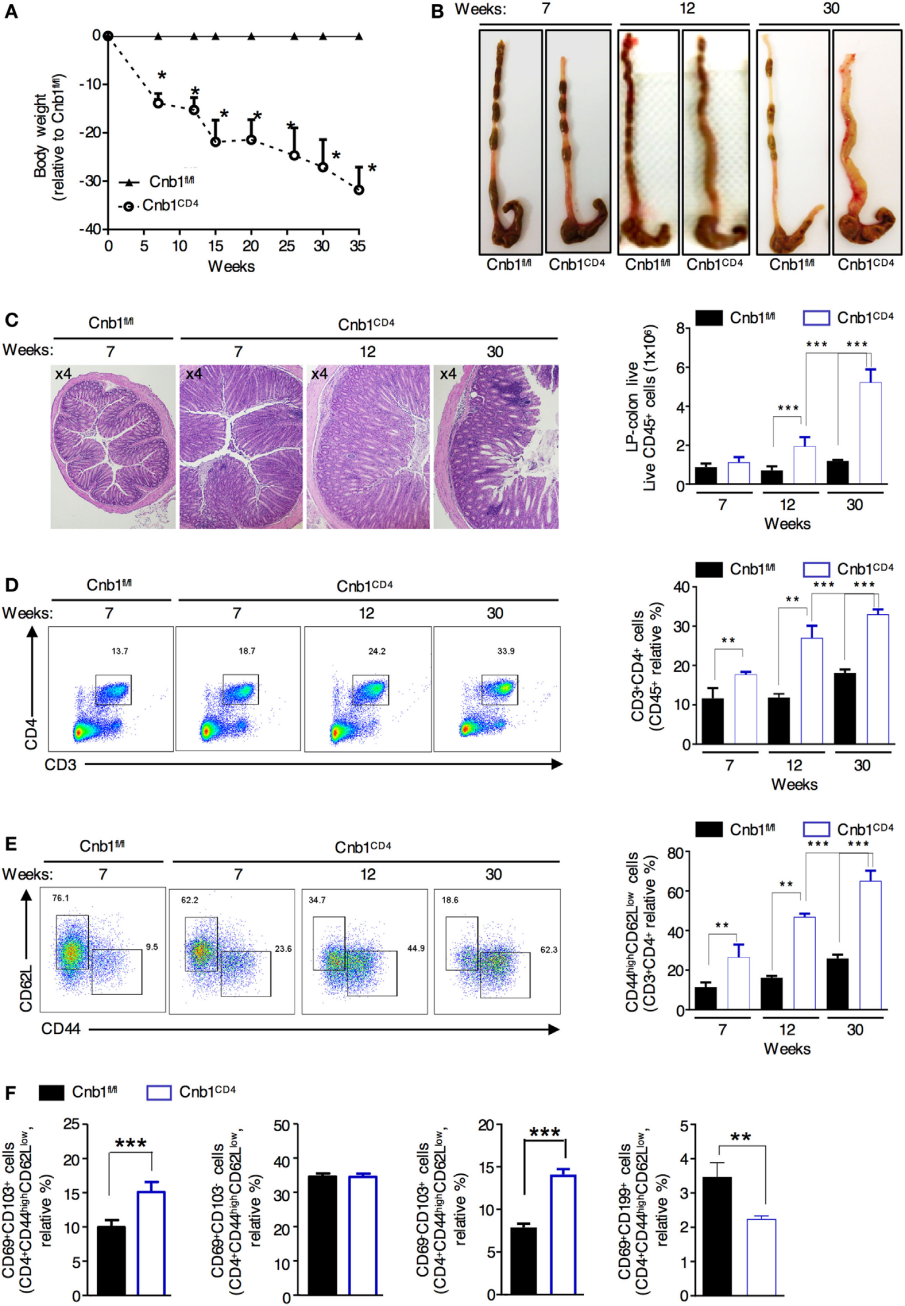
Cnb1^CD4^ mice develop spontaneous colitis. **(A)** Body weight was measured in Cnb1^CD4^ and Cnb1^fl/fl^ mice for 35 weeks and expressed as the weight variation relative to the weight of Cnb1^fl/fl^ control mice. **(B)** Representative macroscopic observations of the colons of Cnb1^CD4^ and Cnb1^fl/fl^ mice aged 7, 12, and 30 weeks. **(C)** Representative histopathologic analysis of the colons stained with hematoxylin and eosin taken from Cnb1^CD4^ mice aged 7, 12, and 30 weeks. Colons are shown at ×4 magnification together with the number of infiltrating leukocytes (far right). **(D,E)** Flow-cytometric analyses showing the proportions of **(D)** CD3^+^CD4^+^ T cells and **(E)** CD44^high^CD62L^neg^ cells among CD4^+^ T cells in the colonic-LP of Cnb1^CD4^ and Cnb1^fl/fl^ mice. Data represent the means ± standard error of four independent experiments (*n* = 4–5 mice per group, per experiment). **P < 0.01; ***P < 0.005 (two-tailed, unpaired Student’s *t*-test). **(F)** Proportion of CD44^high^ CD4^+^ T cells expressing CD69 alone or in combination with CD103 or CD199^+^ CD4^+^ T cells from the colonic-LP of Cnb1^fl/fl^ and Cnb1^CD4^ mice aged 6–8 weeks. Data represent the means ± standard error of two independent experiments (*n* = 2–3 mice per group, per experiment) ***P < 0.005 (ANOVA followed by Tukey’s Multiple Comparison Test). Abbreviation: LP, lamina propria.

At 4 weeks of age, Cnb1^CD4^ mice were indistinguishable from Cnb1^fl/fl^ mice; over time, Cnb1^CD4^ mice exhibited progressive and significant impairment in weight gain resulting in ~35% lower body mass compared to Cnb1^fl/fl^ control mice by 35 weeks of age (Figure 2A). From 5 to 8 weeks of age, Cnb1^CD4^ mice exhibited symptoms of chronic IBD, such as diarrhea. Thickening of the colon wall and shortening of the colon length were also identified from 5 to 8 weeks and worsened by 30 weeks (Figure 2B). Severe, diffuse inflammation was evident throughout the colon and was associated with the loss of mucin and extensive accumulation of mononuclear cells in the mucosa of Cnb1^CD4^ mice compared to that of Cnb1^fl/fl^ mice (Figure 2C; Figure S2A in Supplementary Material).

Flow-cytometric analysis revealed a marked and progressive expansion of total and antigen-experienced CD44^high^CD62L^neg^ CD4^+^ T cells in the colonic-LP of Cnb1^CD4^ mice compared to that of Cnb1^fl/fl^ mice (Figures 2D,E). Moreover, intestinal CD4^+^ T cells also upregulated the expression of either CD39 or CD73, suggesting an increased memory-like phenotype (data not shown) (26, 27). We then asked whether this increase in antigen-experienced CD44^+^ CD4^+^ T cells was due to the expansion of LP-resident memory T cells or the migration of circulating cells into the gut mucosa (28, 29). We observed a preferential expansion of gut-resident CD103^+^CD44^high^CD62L^neg^ CD4^+^ T cells and fewer migratory CD69^+^CD199^+^ (CCR9) CD4^+^ T cells in the LP of Cnb1^CD4^ mice compared to that of Cnb1^fl/fl^ mice (Figure 2F), indicating the prominent expansion of activated gut resident CD4^+^ T cells. Although CD8^+^ T cells expressed increasingly higher levels of CD44 over time, these cells did not expand (Figure S2B in Supplementary Material). Moreover, the composition of the T-cell compartment in the liver was normal (Figure S2C in Supplementary Material). These data suggest that Cnb1 expression in CD4^+^ T cells keeps the effector T-cell population in check, thereby preventing T-cell-mediated destruction of the intestinal tissue.

### Colitis Susceptibility in Cnb1^CD4^ Mice Is Mediated by the Intestinal Microbiota

**Figure 3:**
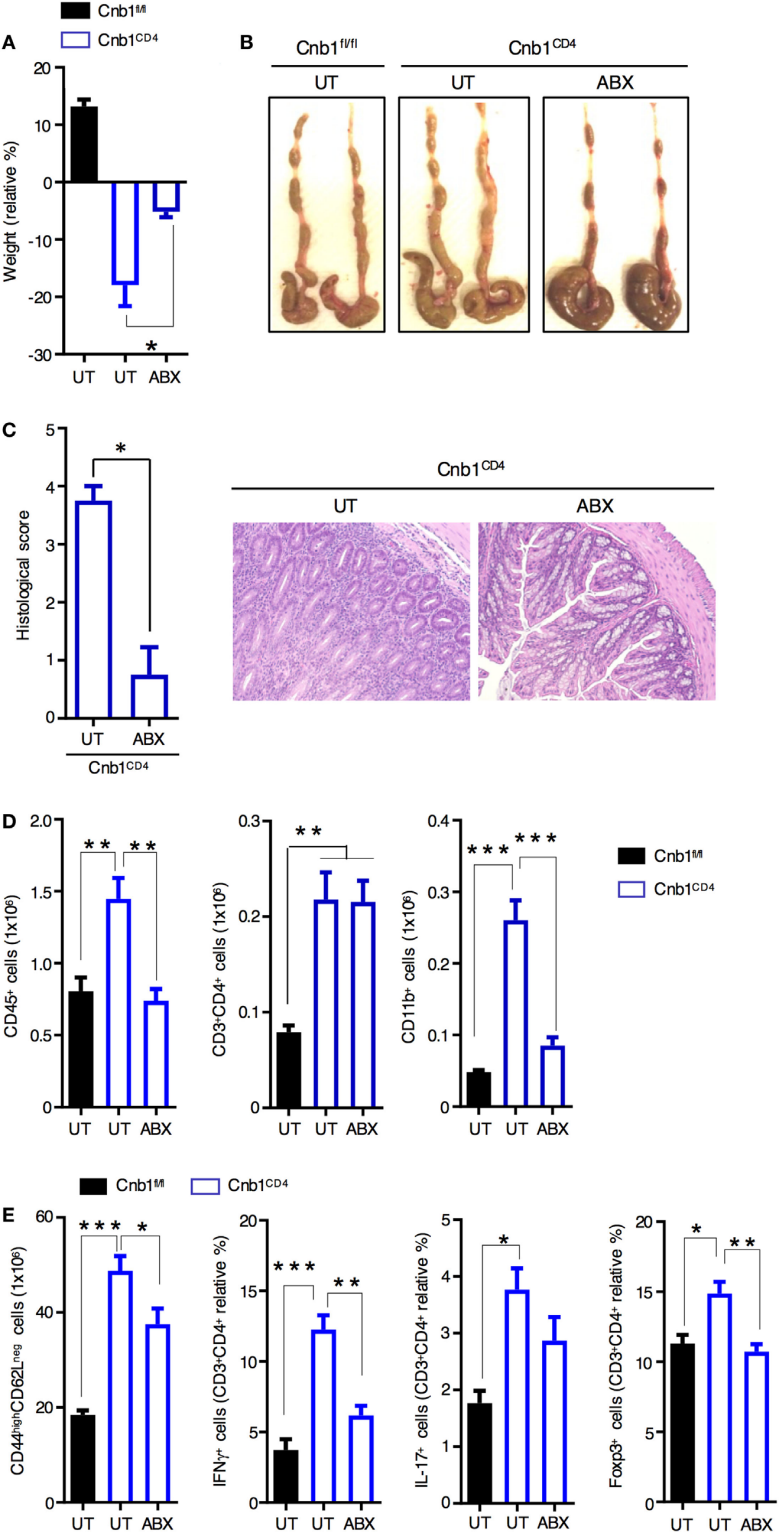
Spontaneous intestinal inflammation is driven by the microbiota in Cnb1^CD4^ mice. **(A)** Change in body weight in untreated (UT) Cnb1^fl/fl^ mice and Cnb1^CD4^ mice treated or not with antibiotics (ABX), from 4 weeks relative to baseline. **(B)** Macroscopic appearance of the colon in UT Cnb1^fl/fl^ mice and Cnb1^CD4^ mice treated or not with ABX for 4 weeks. **(C)** Histological inflammation index of colon sections and representative hematoxylin and eosin staining (×10 magnification) of the colonic-lamina propria of ABX-treated Cnb1^CD4^ mice. **(D)** Absolute number of total leukocytes, CD4^+^ T cells, and CD11b^+^ cells infiltrating the colon of ABX-treated Cnb1^CD4^ mice. **(E)** Frequency of antigen-experienced CD44^high^ CD4^+^ T cells secreting IFNγ or IL-17, or expressing the regulatory T-cell marker Foxp3^+^ obtained from Cnb1^fl/fl^ UT mice and Cnb1^CD4^ mice treated or not with ABX for 4 weeks. *P < 0.05; **P < 0.01; ***P < 0.005 (ANOVA followed by Tukey’s Multiple Comparison Test). Data represent the means ± standard error of two independent experiments (*n* = 2–3 mice per group, per experiment).

Clinical and experimental observations in animal models indicate that intestinal commensal bacteria are involved in the initiation and amplification of IBD (30). We next sought to examine whether the intestinal microbiota contributes to the exacerbated colitis caused by the expanded IFNγ-producing CD4^+^ T-cell population in Cnb1^CD4^ mice. We treated 10-week-old colitic Cnb1^CD4^ mice with full-spectrum antibiotics (ABX) for 4–5 weeks. Weight loss was not evident at 4 weeks in ABX-treated Cnb1^CD4^ mice (Figure 3A), and the colons exhibited a grossly normal appearance compared to untreated Cnb1^fl/fl^ mice (Figure 3B). The cecum was slightly enlarged due to loss of microbiota (Figure 3B), as previously reported (31). Histopathological analyses identified a significant decrease in leukocyte infiltration in the colonic mucosa, and mucus production was restored in the epithelial layer of ABX-treated Cnb1^CD4^ mice after 4 weeks (Figure 3C).

Analysis of the immune-cell composition in the colonic-LP of ABX-treated Cnb1^CD4^ mice revealed normalization of total leukocytes and CD11b^+^ myeloid cells (Figure 3D; Figure S3 in Supplementary Material). Although the number and percentage of total CD4^+^ T cells remained high following ABX treatment, effector CD44^high^CD62L^neg^ CD4^+^ T cells were significantly decreased in Cnb1^CD4^ mice (Figure 3E). ABX treatment drastically reduced the percentage of CD4^+^ T cells producing IFNγ, and in part IL-17, and normalized Foxp3^+^ Treg-cell number (Figure 3E). CD8^+^ T cells, which were slightly reduced in Cnb1^CD4^ mice, marginally expanded upon ABX treatment (Figure S3 in Supplementary Material).

Taken together, these findings indicate that microbiota antigens trigger CD4^+^ T-cell activation and expansion in Cnb1^CD4^ mice, which is causative of the colitic phenotype. These data also suggest that once CD4^+^ T cells are activated and become memory cells, eliminating microbiota antigens reduces the production of T-cell effector cytokines, which helps ameliorate colitis by reducing innate-cell recruitment and mucosal damage (32, 33).

### Expansion of Memory-Like Intestinal CD4^+^ T Cells Is Not Due to Perturbation of Homeostasis or Suppressive Activity of Treg Cells in the Gut

**Figure 4:**
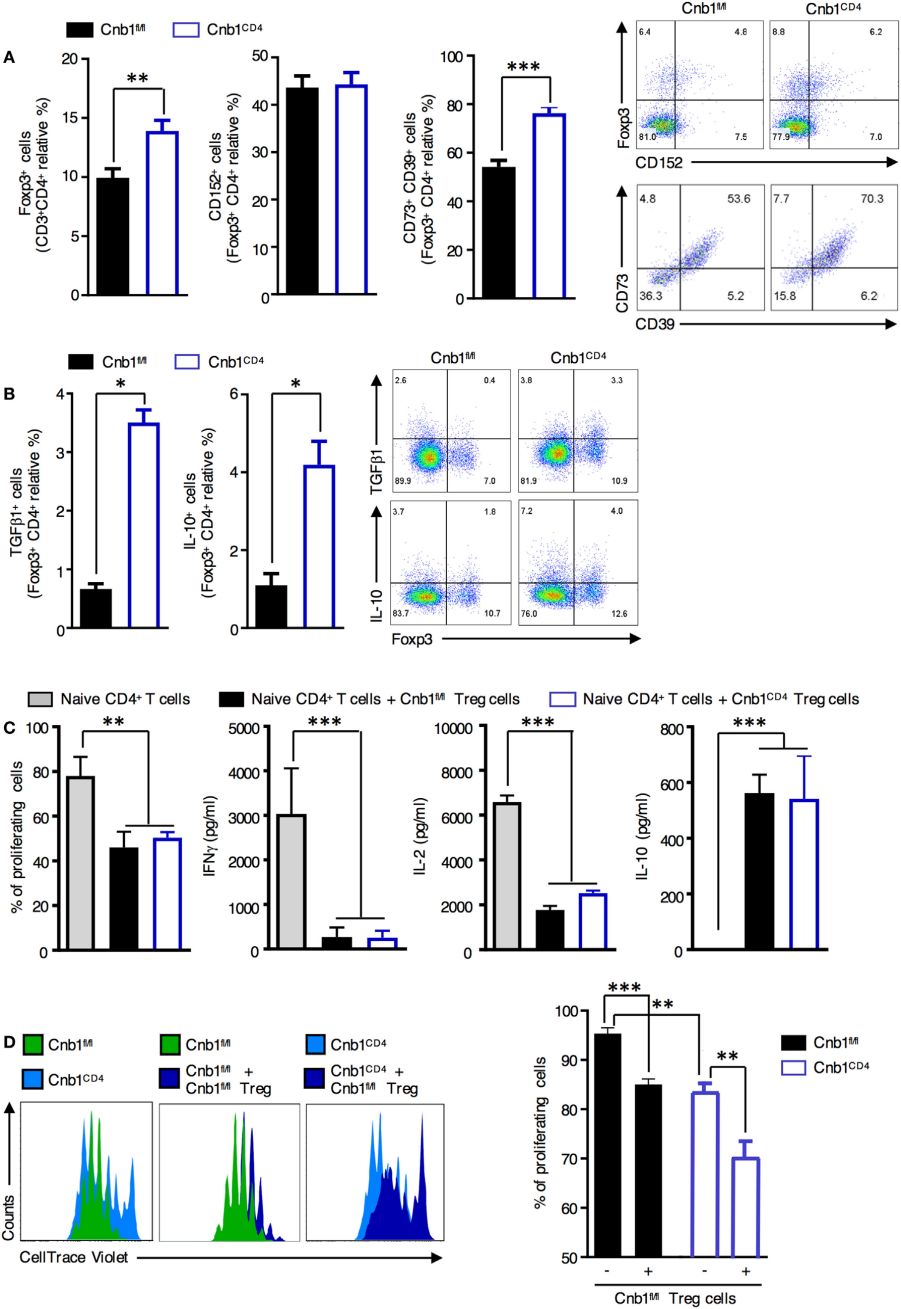
Normal suppressive activity of Treg cells from Cnb1^CD4^ mice. **(A)** Expression patterns of the Treg-cell markers CD152 and CD73/CD39 in FoxP3^+^ CD4^+^ T cells obtained from colonic LP of Cnb1^fl/fl^ and Cnb1^CD4^ mice. Representative dot-plots of the proportions of CD152^+^ and Foxp3^+^ gated on CD4^+^ T cells and CD73^+^ or CD39^+^ gated on Treg cells are shown. **(B)** IL-10 and TGFβ production by Foxp3^+^ and Foxp3^−^ CD4^+^ T cells in colonic-LP of Cnb1^fl/fl^ and Cnb1^CD4^ mice. **(C)** Flow cytometry-based Treg-cell suppression assay. A total of 1 × 10^5^ wild-type splenic-naïve CD4^+^ T cells labeled with CellTrace Violet were activated with anti-CD3 (5 µg/ml), anti-CD28 (2 µg/ml) antibodies, IL-2 (200 U/ml), and dendritic cells (2 × 10^4^) and cultured with or without 1 × 10^5^ Treg cells isolated from the spleens of Cnb1^fl/fl^ and Cnb1^CD4^ mice. After 3 days, the proliferation of responder CD4^+^ T cells was analyzed using FlowJo and expressed as the percentage of proliferating cells. Cytokine production in the culture supernatants was assessed by ELISA. Data represent the means ± standard error of two independent experiments (*n* = 2–3 mice per group, per experiment; cells plated in triplicate). The number of depicted cells ranges between 6,000 and 20,000. **P < 0.01; ***P < 0.005 (ANOVA followed by Tukey’s Multiple Comparison Test). **(D)** Treg cells were sorted by florescence-activated cell sorting from the spleens of Cnb1^fl/fl^ mice and cocultured with CellTrace Violet-labeled-naïve CD4^+^ T cells isolated from the spleens of Cnb1^fl/fl^ and Cnb1^CD4^ mice in the presence of splenic-dendritic cells at a ratio of 5:5:1 (Treg cells:naïve CD4^+^ T cells:dendritic cells), and stimulated with anti-CD3 and anti-CD28 antibodies and IL-2 (200 U/ml) for 3 days. The graph (right) shows the percentage of proliferating cells based on CellTrace Violet dilution, in the presence or absence of Cnb1^fl/fl^ Treg cells, as determined by flow cytometry. Data represent the means ± standard error of two independent experiments (*n* = 2–3 mice per group, per experiment; cells plated in triplicate). **P < 0.01; ***P < 0.005 (ANOVA followed by Tukey’s Multiple Comparison Test). Abbreviation: Treg, regulatory T-cell.

Regulatory T cells are critical for maintaining the gut immune system by helping prevent unrestricted expansion of effector T cells and subsequent immune-mediated pathologies. We found that Foxp3^+^ Treg cells, particularly those coexpressing CD73 and CD39 (involved in cell-to-cell contact-mediated inhibition), were significantly increased in the colonic-LP of Cnb1^CD4^ mice at 7–9 weeks compared to those of controls (Figure 4A). Proliferating Ki-67^+^ Foxp3^+^ Treg cells and Helios^+^ Foxp3^+^ Treg cells were also increased compared to those of controls (Figure S4A in Supplementary Material). Conversely, the total Foxp3^+^ Treg cells in the thymus, spleen, and ILN were diminished in Cnb1^CD4^ compared to those in controls (Figure S4B in Supplementary Material). Cnb1^CD4^ splenic Foxp3^+^ Treg cells expressed CTLA-4 (CD152) and CD73/CD39 at normal levels (Figure S4C in Supplementary Material). TGFβ1- and IL-10-producing Treg cells were increased in the colonic-LP of Cnb1^CD4^ mice (Figure 4B). These data show that Treg cells producing soluble and contact-mediated-suppressive mediators expanded in the intestine, while their pool decreased in the thymus and spleen of Cnb1^CD4^ mice compared to those of Cnb1^fl/fl^ mice.

We next investigated whether Cnb1 deficiency affected Treg-cell-mediated suppression of CD4^+^ T-cell proliferation. CellTrace Violet-labeled-naïve CD4^+^ T cells (CD45RB^high^/CD62L^+^) were incubated with splenic Treg cells (CD45RB^low^CD25^+^GiTR^+^) isolated from either Cnb1^CD4^ or Cnb1^fl/fl^ mice, and T-cell proliferation in response to CD3/CD28 stimulation was assessed after 4 days. Cnb1-deficient Treg cells retained their capacity to suppress CD4^+^ T-cell proliferation, indicating that Cnb1 deficiency does not impact on Treg-cell-mediated immune suppression (Figure 4C). We also determined whether Cnb1-deficient CD4^+^ T cells are sensitive to Treg-cell-mediated suppression. CellTrace Violet-labeled-naïve CD4^+^ T cells from Cnb1^CD4^ mice or Cnb1^fl/fl^ mice were incubated with wild-type Treg cells. In the absence of Treg cells, naïve CD4^+^ T cells from Cnb1^CD4^ mice expanded less *in vitro* in response to CD3/CD28 stimulation compared to Cnb1-sufficient CD4^+^ T cells (Figure 4D). The proliferation of CD4^+^ T cells from Cnb1^CD4^ mice was markedly suppressed by Treg cells (Figure 4D). These results confirm that the expansion of effector/memory-like CD4^+^ T cells in the colonic-LP of Cnb1^CD4^ mice is not due to defects in the numbers or suppressive activity of Treg cells.

### Cnb1-Deficient Colonic CD4^+^ T Cells Produce High Levels of Th1/Th17 Cytokines

**Figure 5:**
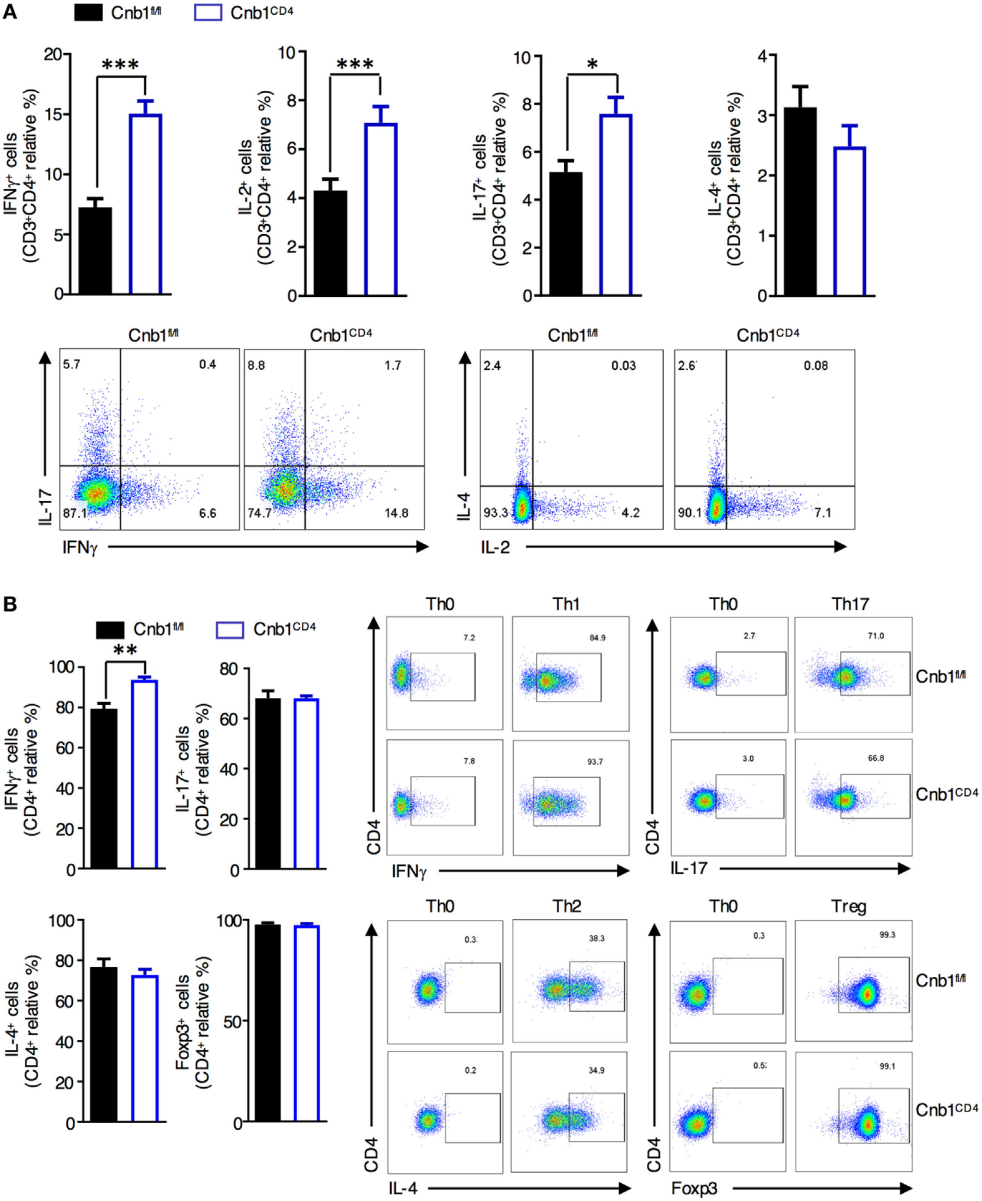
Cnb1-deficient CD4^+^ T cells produce more interferon (IFN)γ. **(A)** Proportion of CD4^+^ T cells producing IFNγ, IL-2, IL-17, or IL-4 in colonic-lamina propria (LP) isolated from Cnb1^fl/fl^ and Cnb1^CD4^ mice. **(B)** Purified naïve CD62L^high^CD44^neg^ CD4^+^ T cells from the spleens of Cnb1^fl/fl^ and Cnb1^CD4^ mice were cultured under conditions to promote Th0, Th1, Th17, Th2, and Treg cells. T cells were collected after 5 days and stimulated with PMA/ionomycin for 5 h, with the addition of Brefeldin A for the final 4 h. Cells were stained for intracellular IFNγ, IL-17, IL-4, and FoxP3, and the proportion of CD4^+^ T cells producing these cytokines was assessed by flow cytometry. Representative flow-cytometric dot plots are shown. Data represent the means ± standard error of two or three independent experiments (*n* = 3–4 mice per group, per experiment). *P < 0.05; **P < 0.01; ***P < 0.005 (two-tailed, unpaired Student’s *t*-test). Abbreviations: Th0, naïve T cell; Th1, Type-1 T helper cell; Th2, Type-2 T helper cell; Th17, T helper 17 cell; Treg, regulatory T-cell.

We next sought to elucidate the mechanisms underlying the increased CD4^+^ T-cell effector function observed in Cnb1-deficient CD4^+^ T cells. Intracellular cytokine staining of CD4^+^ T cells isolated from colonic-LP of Cnb1^CD4^ mice revealed a significant increase in the frequency of T cells producing IFNγ, IL-17, or IL-2, while the fraction of IL-4-producing CD4^+^ T cells was normal (Figure 5A). Cnb1-deficient CD4^+^ T cells exhibited increased activation-induced cell death, as determined by propidium iodide staining and CD95 (Fas) expression analysis, which is indicative of increased activation status and effector function compared to Cnb1-sufficient CD4^+^ T cells (Figure S5 in Supplementary Material).

To determine whether Cnb1-deficient CD4^+^ T cells have an intrinsic defect in polarization along distinct Th lineages, we generated *in vitro*-differentiated Th-cell subsets from splenic-naïve CD4^+^ T cells of Cnb1^CD4^ and Cnb1^fl/fl^ mice. While Cnb1-deficient Th17, Th2, and Treg cells differentiated normally, IFNγ production analysis implied a significant increase in Th1 differentiation from Cnb1-deficient CD4^+^ T cells (Figure 5B). These results indicate that the Cn pathway is a negative regulator of IFNγ production by CD4^+^ T cells.

### Cn Is a CD4^+^ T-Cell-Intrinsic Negative Regulator of the JAK/STAT Pathway

We performed a whole-genome gene expression analysis of CD44^low^ CD4^+^ T cells isolated from the colonic-LP of Cnb1^CD4^ and Cnb1^fl/fl^ mice aged 6–8 weeks to identify the T-cell-intrinsic mechanism(s) responsible for the exacerbated activation of Cnb1-deficient CD4^+^ T cells in the colon (Figure S6 and Supplementary Methods in Supplementary Material). We chose this population of CD4^+^ T cells as these cells exhibit a non-activated phenotype compared to antigen-experienced CD4^+^ T cells that express high levels of CD44 (CD44^high^CD62L^neg^CD103^+^CD69^+^) (28). Indeed, CD44^low^ CD4^+^ T cells from Cnb1^CD4^ mice expressed similar levels of the T-cell activation markers CD199, CD103, and CD69 compared to CD4^+^ T cells isolated from Cnb1^fl/fl^ control mice (Figure S7 in Supplementary Material), indicating that CD44^low^ CD4^+^ T cells from Cnb1^CD4^ mice are yet to receive activatory, inflammatory signals from the inflamed colon mucosa.

**Figure 6:**
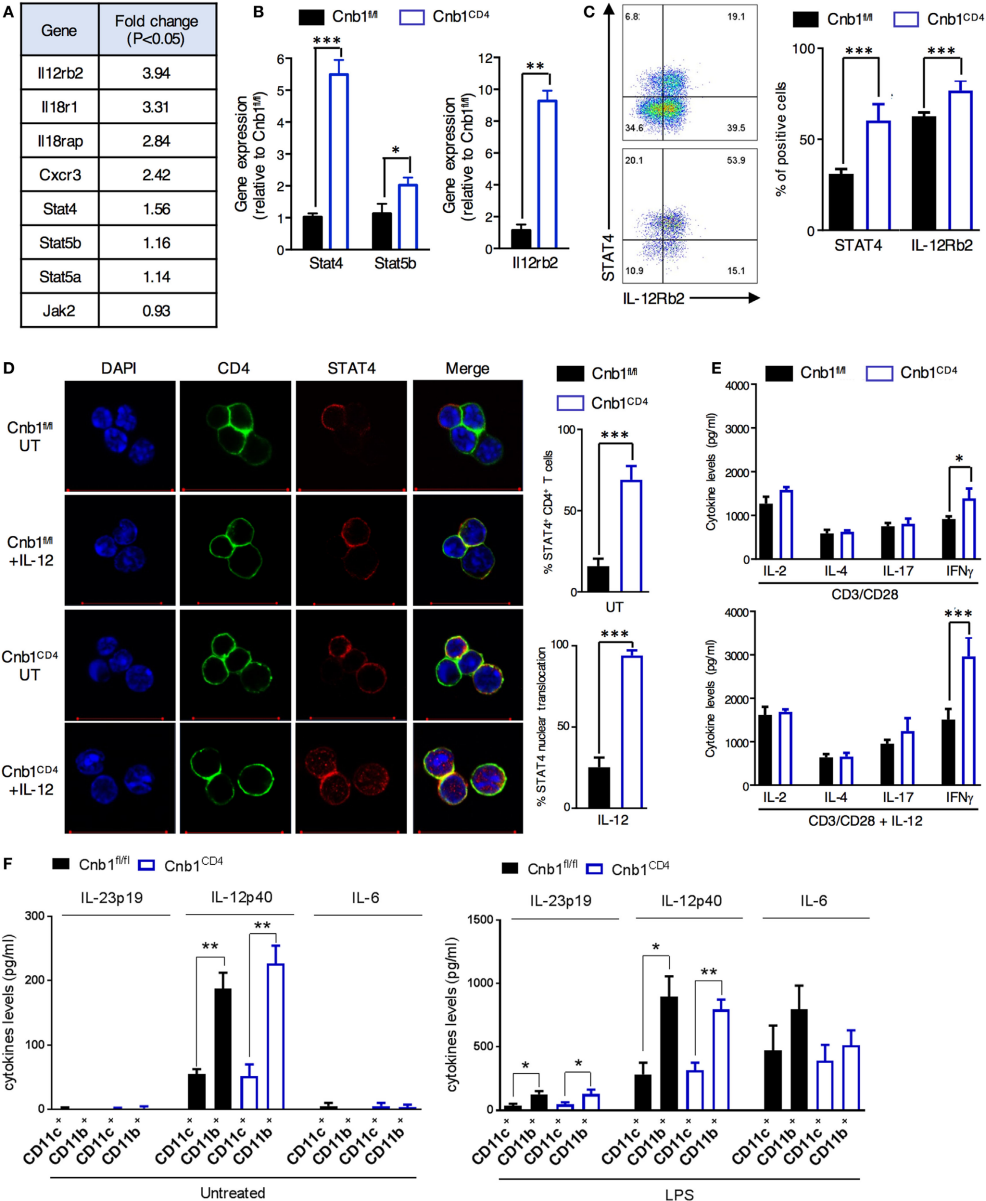
Increased JAK/STAT4 pathway in colonic Cnb1-deficient CD4^+^ T cells. **(A)** Table of some of the differentially expressed genes in CD44^low^ CD4^+^ T cells sorted from colonic-lamina propria (LP) of Cnb1^CD4^ and Cnb1^fl/fl^ mice aged 7–10 weeks. **(B)** Quantitative RT-PCR analysis of mRNA expression of *STAT4, STAT5b*, and *Il-12rb2* in CD44^low^ CD4^+^ T cells sorted from the colonic-LP of Cnb1^CD4^ and Cnb1^fl/fl^ mice. **(C)** Relative expression of STAT4 and IL-12Rβ2 by CD44^low^ CD4^+^ T cells sorted from the colonic-LP of Cnb1^CD4^ and Cnb1^fl/fl^ mice aged 8 weeks, as assessed by flow cytometry. Representative dot plots are shown. Values represent the means ± standard error of two independent experiments (*n* = 2–3 mice per group, per experiment). **(D)** Representative confocal images of STAT4 immunofluorescent staining (red) in CD44^low^ CD4^+^ T cells sorted from the colonic-LP of Cnb1^CD4^ and Cnb1^fl/fl^ mice aged 7–10 weeks old left untreated (UT) or stimulated by recombinant IL-12 (100 ng/ml) for 5 h. The total STAT4 expression and relative nuclear translocation were assessed by confocal microscopy. **(E)** Colonic-LP CD44^low^ CD4^+^ T cells from Cnb1^fl/fl^ and Cnb1^CD4^ mice were stimulated with plate-bound anti-CD3 (5 µg/ml) and soluble anti-CD28 (2 µg/ml) alone or in combination with rIL-12 (100 ng/ml) for 8 h, and culture supernatants were analyzed by ELISA. **(F)** Cytokine production from CD11c^+^ and CD11b^+^ myeloid cells isolated from the mesenteric lymph node of Cnb1^CD4^ and Cnb1^fl/fl^ mice was assessed at steady state and after LPS restimulation (10 µg/ml) by ELISA. *P < 0.05; **P < 0.01, ***P < 0.001 (two-tailed, unpaired Student’s *t*-test). Data represent the means ± standard error of two independent experiments (*n* = 2–3 mice per experiment).

Differential gene expression analysis found that >300 genes were differentially upregulated and ~200 genes were downregulated in Cnb1-deficient CD4^+^ T cells compared to those in Cnb1-sufficient CD4^+^ T cells (Figure S7 in Supplementary Material). A set of cytokine and receptor genes mainly associated with Th1-cell lineage commitment (34), including IL-12Rβ2, IL-18R1, IL-18RAP, and CXCR3 (Figure 6A), were among the most upregulated genes (*P-*value <0.01). Ingenuity pathway analysis of the upregulated genes in Cnb1-deficient CD44^low^ CD4^+^ T cells revealed an enrichment of genes encoding proteins of the JAK/STAT pathway, including STAT4, STAT5a/b, and JAK2 (*P* = 2.77 × 10^−2^). JAK2 transduces IL-6 and IL-12/IL-23p40 receptor signaling *via* STAT4 activation, which enhances the expression of the Th1 cytokines, including IFNγ and IL-2 (33). Our gene expression data were validated in florescence-activated cell-sorted colonic CD44^low^ CD4^+^ T cells from Cnb1^CD4^ and Cnb1^fl/fl^ mice. We found that CD44^low^ CD4^+^ T cells of Cnb1^CD4^ mice expressed higher levels of STAT4, STAT5b, and IL-12Rβ2 at both the mRNA and protein levels (Figures 6B–D; Supplementary Methods in Supplementary Material).

Increased nuclear translocation of STAT4 caused by IL-12 stimulation was observed in Cnb1^CD4^ CD4^+^ T cells isolated from MLN compared to that in Cnb1^fl/fl^ CD4^+^ T cells (Figure 6D). This effect resulted in higher IFNγ production by Cnb1^CD4^ CD4^+^ T cells (~1,500 pg/ml) in response to stimulation with CD3/CD28 alone and to an even higher level (~3,000 pg/ml) with CD3/CD28 combined with IL-12 compared to Cnb1^fl/fl^ CD4^+^ T cells (Figure 6E). Amplified differentiation of CD4^+^ T cells into IFNγ-producing cells could not be explained on the basis of differential IL-12 production, as this cytokine, as well as IL-23p19 and IL-6, was equivalently produced by CD11c^+^ and CD11b^+^ myeloid cells under steady-state conditions and after LPS restimulation *in vitro* (Figure 6F). Taken together, these findings suggest that Cnb1 in CD4^+^ T cells restrains intestinal inflammation by negatively controlling the STAT4 pathway in response to IL-12.

## Discussion

The contribution of the Ca^2+^–Cn–NFAT-signaling pathway to T-cell differentiation, activation, and tolerance has been extensively studied (2, 3, 35), but the involvement of Cn-mediated signaling in the establishment, maintenance, and regulation of immune functions in CD4^+^ T cells of the intestinal tract is largely unknown. Here, we generated a conditional mouse knockout of CnB in CD4^+^ T cells to demonstrate the central role of Cn in intestinal homeostasis. Mutant animals exhibited marked colonic inflammation, supported by differences at the molecular level in terms of the phenotypes of CD4^+^ T cells and the T-cell-derived cytokines produced in the colonic mucosa compared to controls. Specifically, tissue-resident Cn-deficient CD4^+^ T cells expanded preferentially in the colonic mucosa and produced markedly high levels of IFNγ and IL-2, thereby causing inflammation in the colon.

T-cell receptor engagement activates various signaling pathways that lead to the differentiation of CD4^+^ T cells. One of these signaling cascades culminates in the Cn-mediated translocation of NFAT to the nucleus. All NFATs, except NFAT3, are expressed in peripheral lymphocytes: NFAT4 is preferentially expressed in thymocytes, whereas NFAT1 and NFAT2 are mainly expressed in peripheral T cells (2). Double NFAT1^−/−^NFAT4^−/−^mice exhibited impaired positive selection of naïve CD4^+^ T cells and the development of natural Treg cells but normal Treg-cell-suppressive activity (25). Similarly, we found that Cnb1 deletion in CD4^+^ T cells affected the balance between CD4^+^ T cells and nTreg cells, but not the inhibitory activity of Treg cell, confirming the requirement of Ca^2+^ signals in nTreg-cell development (20) and that NFAT activity is not required for the suppressor activity of Treg cells *in vitro* and *in vivo* (25, 36). Instead, the induction of iTreg cells occurs primarily in gut-associated lymphoid tissues, where iTreg cells balance Th17-driven immune responses *via* TGFβ production dependently on NFAT expression (25). Here, we found that Foxp3^+^ Treg cells from Cnb1^CD4^ mice expanded in the inflamed intestinal mucosa and upregulated the production of immunomodulatory cytokines (TGFβ and IL-10) and the expression of surface molecules (CD73 and CD39), which are crucial for the suppression of effector T cells. However, it appears that despite an increase in Treg-cell pool in the intestine probably due to the presence of inflammation in this model, the expansion of memory-like (effector) T cells in the intestine and the overproduction of pathogenic effector cytokines as a result of Cnb deletion seem not to be contained.

The most overt phenotype of our Cnb1^CD4^ mice was severe colitis associated with the vigorous expansion of effector CD4^+^ T cells in the intestine. Loss of Cnb1 in intestinal CD4^+^ T cells induced the upregulation of many genes involved in T-cell homeostasis and effector function, indicating a reprogramming of these cells in the absence of Cn–NFAT signaling. The subsequent loss of NFAT activation led to robust, increased activation of STAT4 and predisposed these cells to differentiate toward a Th1-cell phenotype. Indeed, we found that JAK2 and STAT4 together with genes involved in promoting Th1-cell activity (including Cxcr3, Il-18r1, Il-18rap, and Il-12rb2) were also upregulated in CD4^+^ T cells from Cnb1^CD4^ mice compared to those from controls.

As discussed, we identified an intrinsic predisposition of Cnb1-deficient CD4^+^ T cells to become Th1 effectors. First, the loss of Cnb1 expression in CD4^+^ T cells resulted in increased *in vitro* differentiation into effector Th1 cells, while Th17, Th2, and Treg-cell differentiation was normal. Second, IL-12R and STAT4 were expressed at higher levels in colonic CD44^low^ CD4^+^ T cells. Third, CD4^+^ T cells from Cnb1^CD4^ mice released higher amounts of IFNγ in response to TCR stimulation by anti-CD3/CD28 compared to those from controls, which was further increased in the presence of IL-12.

In experimental mouse models, STAT4 hyperactivation results in the spontaneous development of transmural colitis, which is a similar condition to CD in human patients (37) and consistent with our Cnb1^CD4^ model where we observed an expansion of IFNγ-producing CD4^+^ T cells in response to commensal antigens. Interestingly, STAT4 and Th1-secreted cytokines, such as IFNγ and IL-2, are critical for the pathogenesis of CD but not UC (38). Rather, UC is associated with CD1d-restricted nonclassical natural killer T cells secreting Th2-cell-associated cytokines, such as IL-13 (9).

The cytokine milieu produced by intestinal myeloid cells, including dendritic cells and macrophages, facilitates the differentiation of T cells into subsets with different effector functions. The engagement of cytokine receptors by specific cytokines activates the JAK/STAT pathway leading to specific cellular responses (32, 33). JAK2 is a transducer of IL-6 and IL-12/IL-23p40-receptor signaling *via* STAT4 activation. This pathway helps enhance the expression of Th1 cytokines, including IFNγ and IL-2 (32, 33). Intestinal dendritic cells and macrophages constitutively produce IL-6, IL-23, and IL-12 in response to components of the normal microbiota (39, 40). We found that microbiota removal by antibiotic treatment reduced the activation and expansion of CD4^+^ T cells in Cnb1^CD4^ mice. As myeloid compartment remained intact in these mice, it can be assumed that microbial and autologous antigens are normally presented to Cnb1-deficient CD4^+^ T cells, but it is also possible that these cells mount a pathological immune response, which is causative of the spontaneous colitic phenotype. One possible mechanism is that Cnb1-deficient CD4^+^ T cells exhibit a stronger response to inflammatory cytokines released by myeloid cells, as emerged by our data showing that the continuous suppression of the Cn pathway leads to an exacerbated Th1 response due to an increased activation of the JAK/STAT pathway. Moreover, the Cn inhibitors CsA and tacrolimus, mainly used to suppress T-cell activation in patients with steroid refractory, acute severe UC (41, 42), can also affect other cellular compartments, including myeloid cells (43), as well as other cellular processes (23). Our data suggest that treatments, which target the JAK/STAT pathway, may serve as viable strategies to effectively control IBD. The new oral pan-JAK inhibitor tofacitinib has been developed and is under evaluation in inflammatory and autoimmune disorders, including IBD (44). Although some studies found that tofacitinib is effective and safe in UC, other studies have reported no positive effect in CD (44). Additional studies with larger patient cohorts are warranted before JAK inhibitors can be offered as potential therapeutics for IBD.

Polymorphisms in *IL-23R, IL-12B*, and *JAK2* genes were associated with an increased risk of IBD (45). Furthermore, a meta-analysis of genome-wide association studies in CD identified new susceptibility loci encoding components of innate immunity, T-cell signaling, and epithelial barrier function (46). Up to date, to the best of our knowledge, polymorphisms in the *PPP3R1* gene encoding Cnb have not been identified. However, it should be considered that Cn modulates the activity of not only NFATc but also several other transcription factors, such as NF-κB, AP-1, and Elk1 and also interferes with other signaling pathways, such as TGF-β-dependent signaling and the MAPK cascade (23). Further studies assessing any potential *PPP3R1* gene polymorphism in IBD patients and other gastrointestinal complications could be useful to fully elucidate the mechanism and role of this important signaling pathway on intestinal immunity in humans.

In conclusion, our study has identified an unprecedented and biologically relevant *in vivo* role for Cn as a regulator of the JAK/STAT pathway in CD4^+^ T cells to maintain IL-12-mediated IFNγ production by Th1 cells, thereby preventing intestinal inflammation. Improved understanding of the cross-talk between the Cn–NFAT and JAK/STAT pathways will advance our understanding on intestinal homeostasis, which we anticipate will generate new avenues for clinical intervention that will benefit patients with IBD.

## Ethics Statement

All experimental procedures were approved by the IBC and IACUC of the BRC (A*STAR) in compliance with their Guidelines for Animal Experiments.

## Author Contributions

Conceptualization, AM and AMo; Investigation, AM, MV, HK, and AT; Data curation, EA, FZ, and MP; Writing—Original Draft, AM and AMo; Writing—Review and Editing, MV and HK; Funding Acquisition, AMo; Supervision, AM and AMo.

## Conflict of Interest Statement

The authors declare that the research was conducted in the absence of any commercial or financial relationships that could be construed as a potential conflict of interest.
