# Supplementary material for: Calcineurin B in CD4+ T Cells Prevents Autoimmune Colitis by Negatively Regulating the JAK/STAT Pathway
Source: Front Immunol. 2018 Feb 19;9:261. doi: 10.3389/fimmu.2018.00261 (PMC5826051; doi:10.3389/fimmu.2018.00261)
Supplement: Supplementary file 1 [file presentation_1.PDF]

SUPPLEMENTARY MATERIAL

Calcineurin B in CD4^{+} T cells prevents autoimmune colitis by negatively regulating the JAK/STAT pathway

Andrea Mencarelli, Maurizio Vacca, Hanif Javanmard Khameneh, Enzo Acerbi, Alicia Tay, Francesca Zolezzi, Michael Poidinger, and Alessandra Mortellaro

SUPPLEMENTARY FIGURES

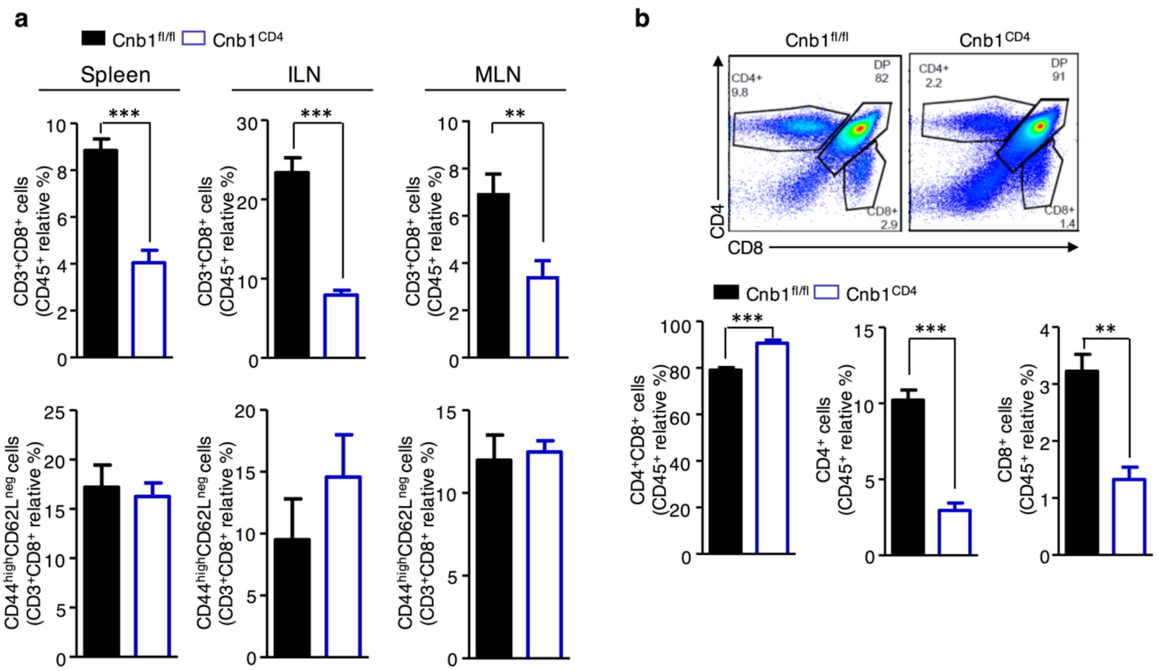

Figure S1. Phenotypic characterization of T cells in peripheral lymphoid organs and thymus of Cnb1^{CD4} mice. (**a**) Proportion of total and CD44^{high}CD62L^{neg} CD8^{+} T cells in spleen, inguinal lymph nodes (ILN) and mesenteric lymph nodes (MLN) of Cnb1^{CD4} and Cnb1$^{fl/fl}$ mice aged 6-8 weeks. (**b**) Percentage of CD4^{+}CD8^{+} double-positive, CD4^{+} and CD8^{+} single positive T cells isolated from the thymus of Cnb1^{CD4} and Cnb1$^{fl/fl}$ mice aged 6-8-weeks. A representative flow cytometric analysis of thymocytes stained for CD4 and CD8 is also shown. Data represent the means ± standard error of two independent experiments (n = 2-4 mice per group, per experiment). **P < 0.01; ***P < 0.005 (two-tailed, unpaired Student's t test).

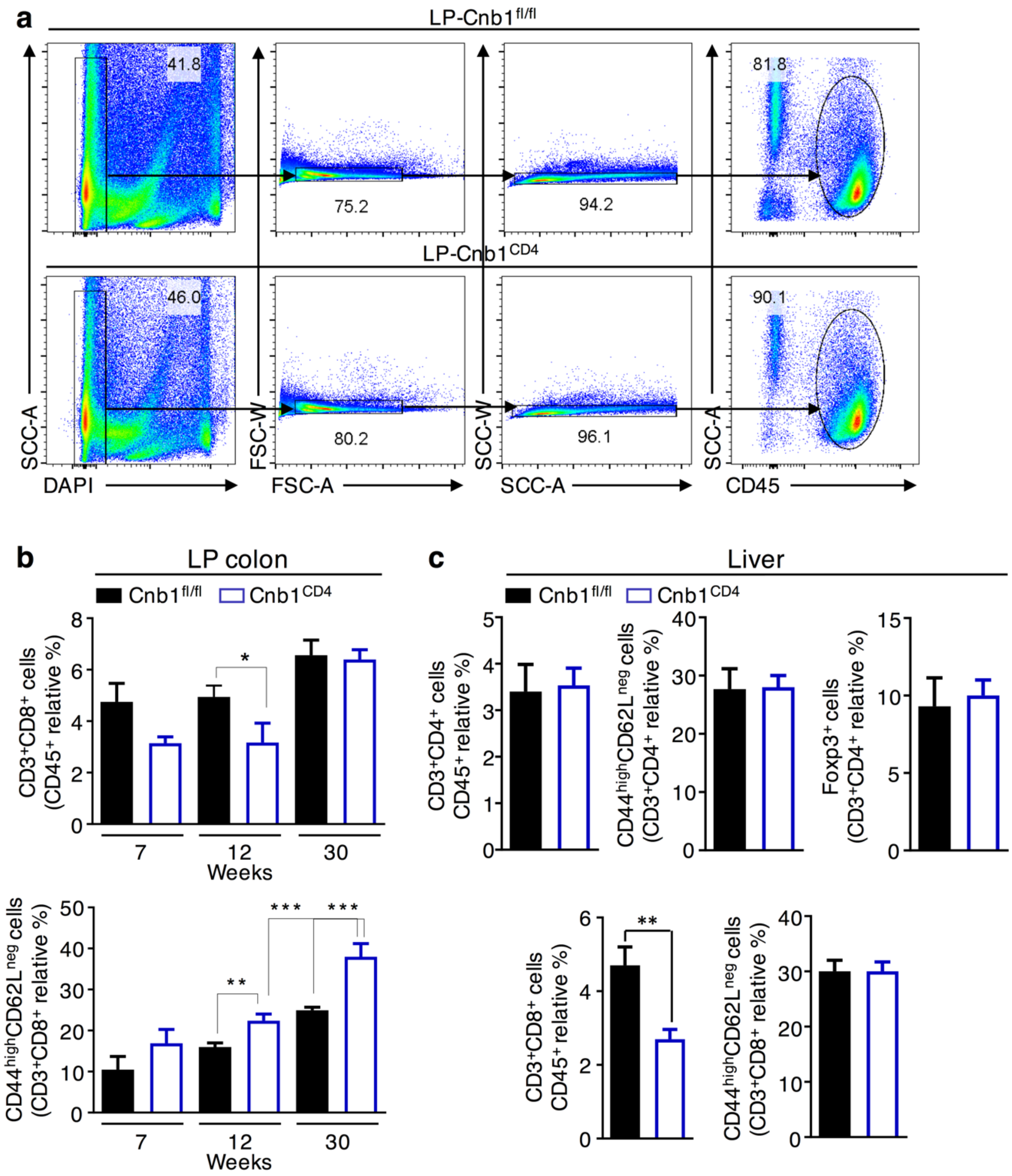

Figure S2. Phenotypic characterization of intestinal and hepatic T cells from Cnb1^{CD4} and Cnb1$^{fl/fl}$ mice. (**a**) Gating strategy for the identification of live (DAPI^{-}) immune cells (CD45^{+}) in cell preparations of colonic lamina propria of Cnb1^{CD4} and Cnb1$^{fl/fl}$ mice. (**b**) Frequency of total and CD44^{high}CD62L^{neg} CD8^{+} T cells in the colonic-lamina propria of Cnb1^{CD4} and Cnb1$^{fl/fl}$ mice aged 6-8-weeks, during the course of colitis. Data show the mean number of lamina propria mononuclear cells obtained from each colon and their characterization based on marker expression. Data represent the means ± standard error of four independent experiments (n = 4-5 mice per group, per experiment). (**c**) Immune phenotype of hepatic T cells obtained from Cnb1^{CD4} mice aged 6-8 weeks compared to control mice. Data represent the means ± standard error of two independent experiments (n = 2-3 mice per group, per experiment). *P < 0.05; **P < 0.01; ***P < 0.005 (two-tailed, unpaired Student's t test).

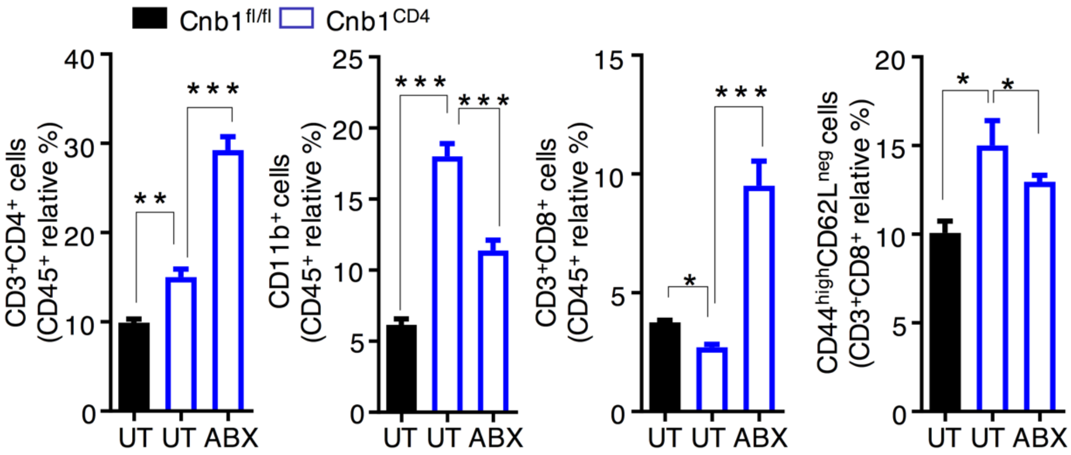

Figure S3. The susceptibility of Cnb1^{CD4} mice to develop colitis depends on the intestinal microbiota. Immune phenotype of colonic-lamina propria of 15-16-weeks old Cnb1^{CD4} mice treated or not with antibiotics (ABX) for 4 weeks compared to untreated (UT) Cnb1$^{fl/fl}$ mice. Data represent the means ± standard error of two independent experiments (n = 2-3 mice per group, per experiment) **P* < 0.05; ***P* < 0.01; ****P* < 0.005 (two-tailed, unpaired Student's t test).

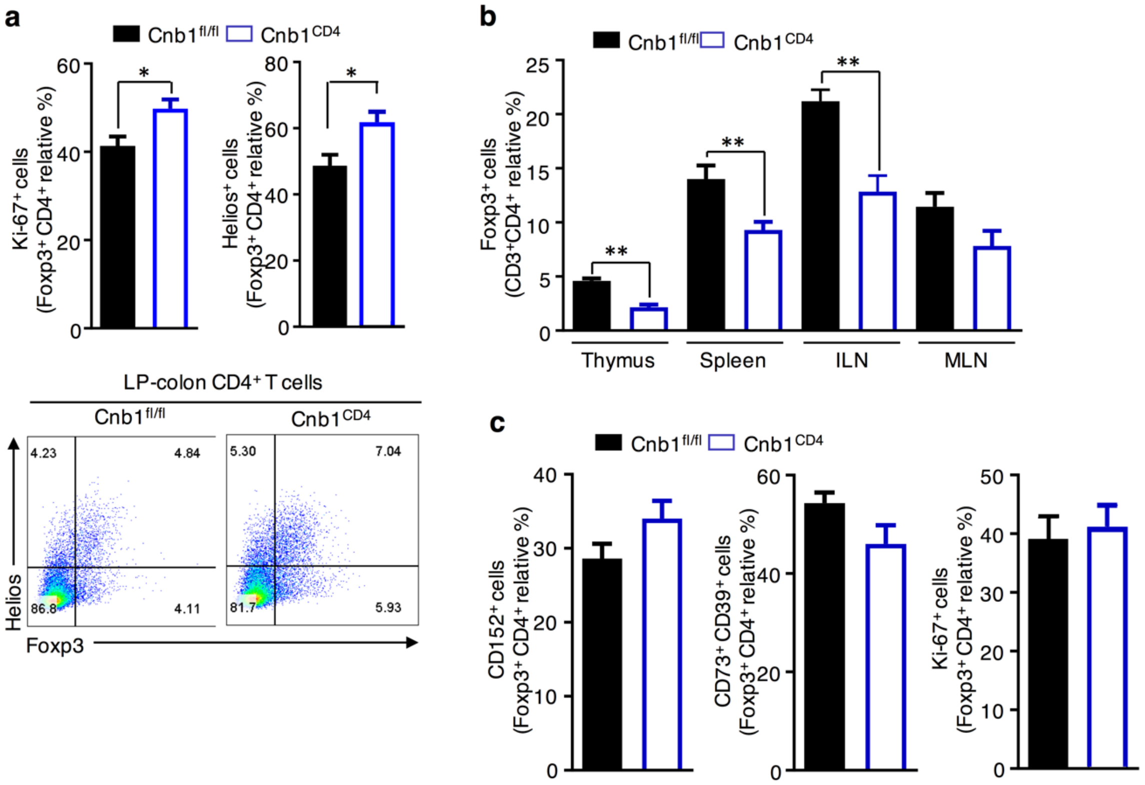

Figure S4. Foxp3$^+$ regulatory T (Treg) cells were found in high proportion in the LP-colon of Cnb1^{CD4} mice. (**a**) Proportion of Ki-67$^+$ and Helios$^+$ in Foxp3$^+$ Treg cells from colonic-lamina propria (LP) of Cnb1^{CD4} and Cnb1$^{fl/fl}$ mice aged 10-13 weeks. Representative figures of flow cytometric analyses are also shown. (**b**) Percentage of Foxp3$^+$ Treg cells in thymus, spleen, inguinal lymph node (ILN) and mesenteric lymph node (MLN) of Cnb1^{CD4} and Cnb1$^{fl/fl}$ mice aged 6-8 weeks. (**c**) Comparison of the proportion of splenic CD152$^+$, CD73$^+$CD39$^+$ and Ki-67$^+$ Foxp3$^+$ Treg cells between Cnb1^{CD4} and Cnb1$^{fl/fl}$ mice. Data represent the means ± standard error of two independent experiments (n = 2-5 mice per group, per experiment) **P* < 0.05; ***P* < 0.01 (two-tailed, unpaired Student's t test).

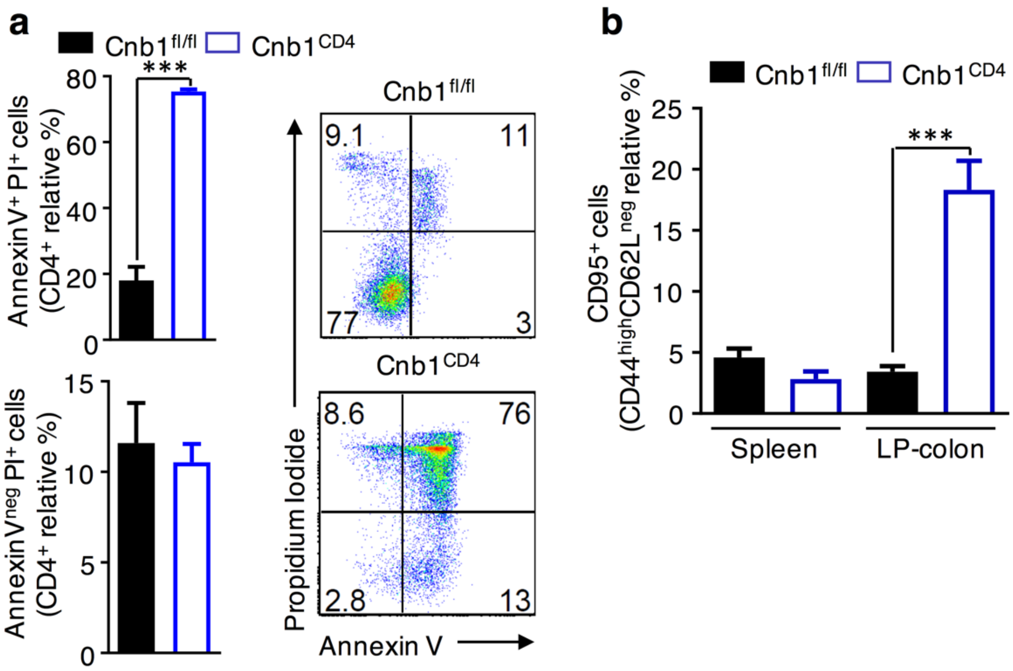

Figure S5. Cnb1-deficient CD4⁺ T cells isolated from the colonic-lamina propria (LP) underwent more activation-induced cell death (AICD) and express CD95/Fas at higher levels than control mice. (**a**) Sorted $CD4^+$ T cells from colonic-LP of $Cnb1^{CD4}$ and $Cnb1^{fl/fl}$ mice aged 10-12 weeks were stimulated *in vitro* with anti-CD3/CD28 antibodies for 24 h, and the proportion of apoptotic and necrotic cells was evaluated by Annexin V and propidium iodide (PI) staining. (**b**) CD95/Fas expression in $CD44^{high}CD62L^{neg}$ $CD4^+$ T cells from spleen and colonic-LP of $Cnb1^{CD4}$ and $Cnb1^{fl/fl}$ mice. Data represent the means ± standard error of either two or three independent experiments (n = 2-3 mice per experiment). ****P* < 0.005 (two-tailed, unpaired Student's t test).

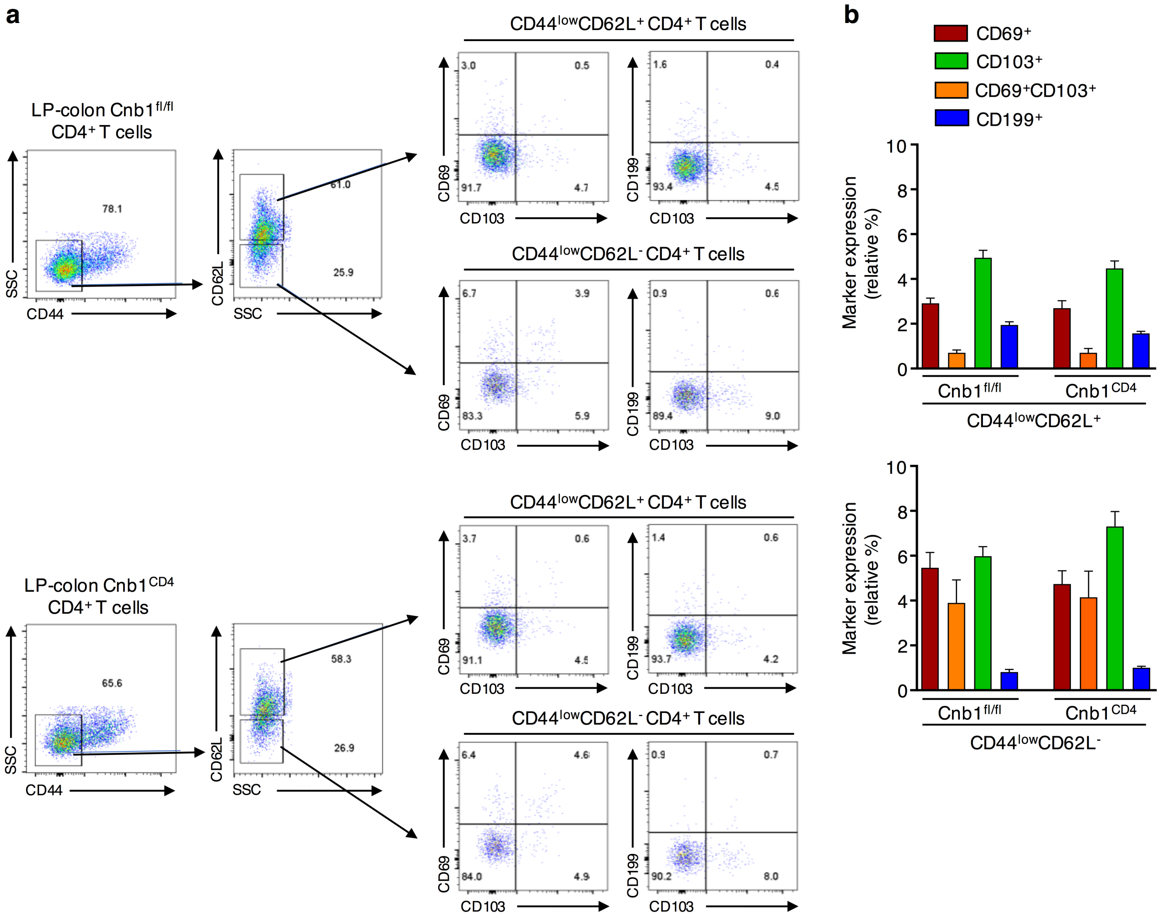

Figure S6. Phenotype of CD4$^+$ T cells colonic-lamina propria (LP) of Cnb1$^{fl/fl}$ and Cnb1^{CD4} mice. (**a**) Gating strategy used to sort CD44^{low} CD4$^+$ T cells used for microarray analysis from colonic-lamina propria (LP) of Cnb1$^{fl/fl}$ and Cnb1^{CD4} mice. (**b**) Proportion of CD4$^+$ T cells expressing the markers CD69, CD103 and CD199 out of CD44^{low}CD62L$^+$ and CD44^{low}CD62L$^-$ cells. Data represent the means ± standard error of either two independent experiments (n = 5-6 mice, 5-7 weeks old, each experiment/group).

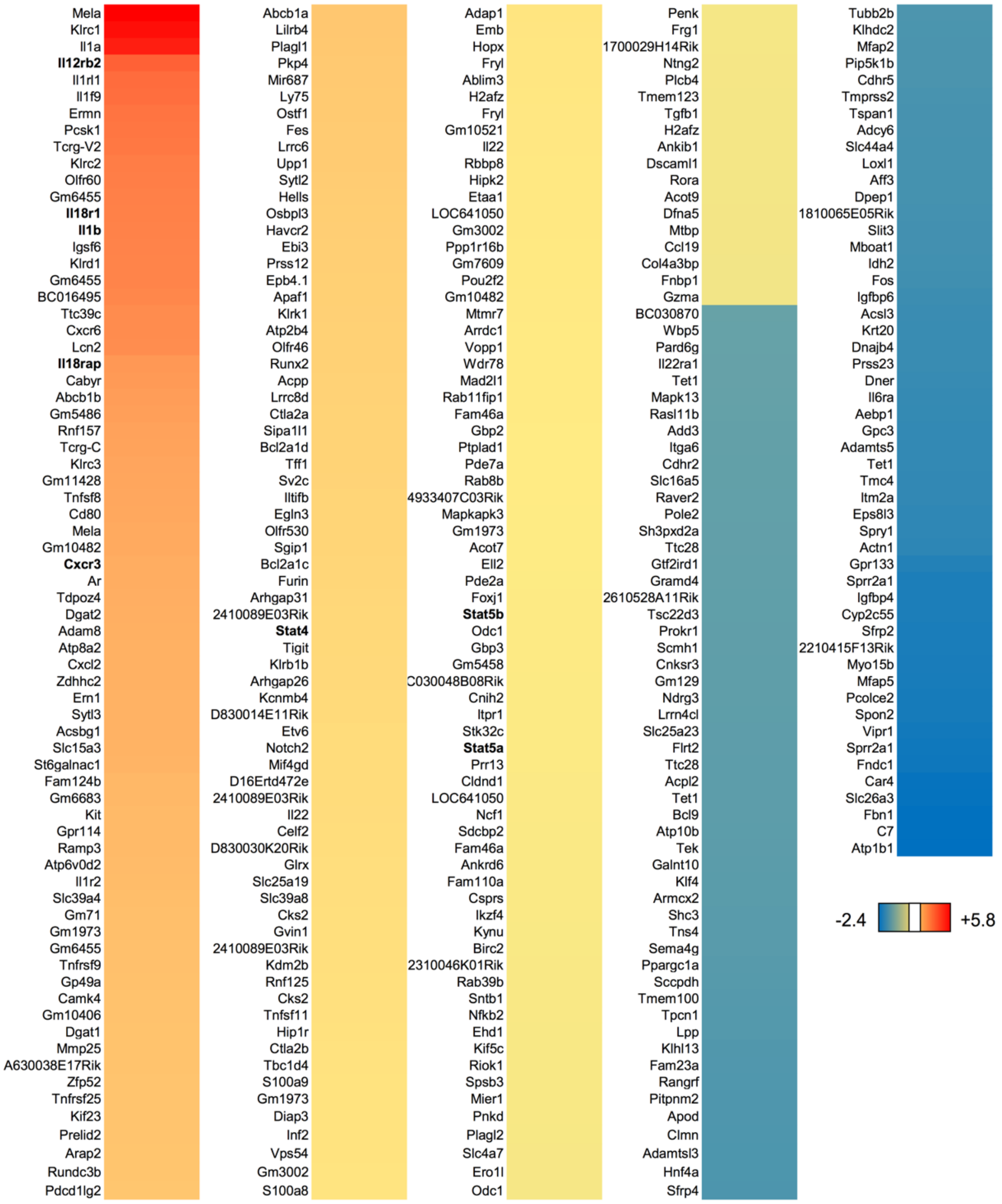

Figure S7. Heat-map of differentially expressed genes in CD44^{low} CD4^{+} T cells from colonic-lamina propria of Cnb1^{CD4} mice compared to Cnb1$^{fl/fl}$ mice.

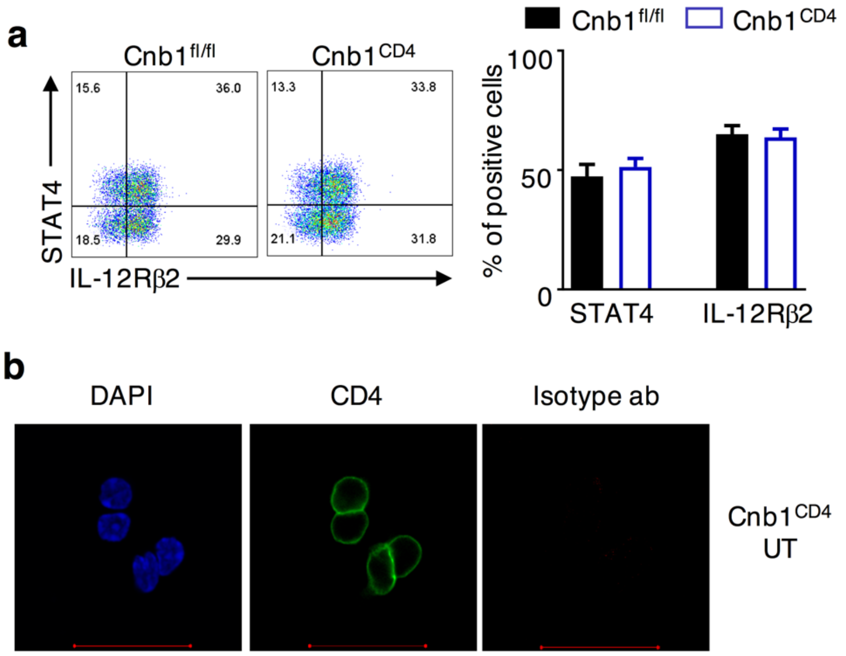

Figure S8. Stat4 and Il12rβ expression in splenic CD44^{low} CD4^{+} T cells of Cnb1$^{fl/fl}$ and Cnb1^{CD4} mice. (**a**) Representative flow cytometric dot plots showing the expression of STAT4 and IL12Rβ2 (left) in splenic CD4^{+} CD44^{low} T cells of Cnb1$^{fl/fl}$ and Cnb1^{CD4} mice aged 8 weeks. Data represent the means ± standard error of two independent experiments are shown on the right (n = 2-3 mice per group, per experiment). (**b**) Representative images of immunofluorescent staining using the isotype control antibody (ab) for STAT4. Abbreviation: UT, untreated.

SUPPLEMENTARY METHODS

Flow cytometry and cell sorting

Cell suspensions were prepared from spleen and colonic-LP and used for flow cytometry or cell sorting the next day. The following anti-mouse antibodies were used at a 1:100 dilution, unless specified otherwise: CD3ε (500A2), CD4 (RM4-5), CD11b (M1/70) CD11c (N418), CD44 (IM7), CD45 (30-F11), CD45R B220 (RA3-6B2), CD45RB (C36-16A), CD62L (MEL-14), CD69 (H1.2F3), CD103 (2E7), I-A/I-E (MHCII, M5/114.15.2), GITR (YGITR 765), CD25 (PC61), CD8 (53-6.7), CD19 (6D5 and EBIO 1D3), CD95 (Jo2), CD73 (TY/11.8), CD39 (Duha59) and CD199 (CW1.2). DAPI was used to distinguish live/dead cells. Origin and catalogue number of all antibodies are listed in Supplementary Table I. For Foxp3 and Helios staining, $CD4^{+}$ T cells were fixed and permeabilized with mouse Foxp3 Staining Buffer Set (BD Bioscience) and stained with Foxp3 (MF23) and Helios (2F6) antibodies (BD Bioscience).

Quantitative real-time PCR

Total cellular RNA was extracted using an Arcturus® PicoPure® RNA Isolation Kit (Thermo Fisher Scientific). Reverse transcription was carried out using high-capacity cDNA Reverse Transcription Kits with RNase Inhibitor (Applied Biosystems), or the SuperScript III First-Strand Synthesis System for RT-PCR (Invitrogen). The following primers were used for RT-PCR: *Gapdh* forward 5'-TCGTCCCGTAGACAAAATGG-3', reverse 5'-TTGAGGTCAATGAAGGGGTC-3'; *Stat4* forward 5'-CCTGGGTGGACCAA TCTGAA-3', reverse 5'-CTCGCAGGATGTCAGCGAA-3'; *Stat5b* forward 5'-GGTCCCCTGTGAGCCCGCAAC-3', reverse 5'-TGACTGTGCGTGAGGGATCCAC TGACT-3'; *Il12rb2* forward 5'-CCTCAATGGTATAGCAGAAC-3', reverse 5'-TAGCCTTGGAATCCTTGG-3'.

Microarray preparation and bioinformatics analysis

RNA extraction was performed using the Arcturus® Picopure® RNA Purification Kit according to the manufacturer's instructions, and RNA was analyzed using an Agilent Bioanalyser for quality assessment. All samples had RNA Integrity Number > 8.5. Amplified sense-strand cDNA was prepared from total RNA (100 ng) according to the Ambion WT Expression Kit instruction manual, and fragmented and labeled using the Affymetrix GeneChip WT Terminal Labeling Kit. Fragmented samples were hybridized for 17 h at 45°C on the GeneChip Mouse Gene 1.0 ST Array according to the manufacturer's instructions. The arrays were then washed and stained using the standard fluidics protocol (FS450_0007) for gene arrays on the GeneChip Fluidics Station 450. The mouse gene chips were scanned using a GeneChip Scanner 3000 and the images were analyzed using Expression Console™ (Affymetrix). Standard array quality-control analyses were carried out according to the manufacturer's instructions.

Affymetrix arrays were normalized with RMA using the R oligo package and differential gene expression was determined using Limma. All microarray data are available from the GEO database under Accession Code GSE101785. Gene ontology analysis was performed on the set of differentially expressed genes using the PANTHER1 platform.[44] Statistical over-representation test for pathways was run and results associated with $P < 0.05$ were considered significant.

SUPPLEMENTARY TABLES

Supplementary Table I. List of antibodies used for flow cytometry

Antibody	Cat. number	Brand
anti-mouse CD3e Pacific blue	558214	BD Pharmingen
anti-mouse CD4 APC	100516	BioLegend
anti-mouse CD4 APC/Cy7	100526	BioLegend
anti-mouse CD4 PE	130310	BioLegend
anti-mouse CD4 eFluor® 450	48-0042-82	eBioscience
anti-mouse CD11b PerCP/Cy5.5	101228	BioLegend
anti-mouse CD11b Alexa Fluor® 700	101222	BioLegend
anti-mouse CD11b APC	17-0112-83	eBioscience
anti-mouse/human CD11b PE	101208	BioLegend
anti-mouse CD11c PE/Cy7	117318	BioLegend
anti-mouse CD11c PE	117307	BioLegend
purified anti-mouse CD16	553142	BD Pharmingen
anti-mouse CD25 PE	130-102-593	Miltenyi Biotec
anti-mouse CD44 PECy7	103030	BioLegend
anti-mouse CD45 APC	103112	BioLegend
anti-mouse CD45 APC/Cy7	103116	BioLegend
anti-mouse CD45 Alexa Fluor 700	103128	BioLegend
anti-mouse CD45 FITC	103107	BioLegend
anti-mouse CD62L Antibody FITC	553150	BD Pharmingen
anti-mouse CD19 PerCP/Cy5.5	115533	BioLegend
anti-mouse CD19 FITC	152404	BioLegend
anti-mouse CD69 APC	104514	BioLegend
anti-mouse CD103 Biotin	121404	BioLegend
anti-mouse I-A/I-E APC/Cy7	107628	BioLegend
anti-mouse FoxP3 AF488	560403	BD Biosciences
anti-mouse CD357 (GITR) PE-Cy7	120222	BioLegend
anti-mouse CD95 PE-CY7	557653	BD Biosciences
anti-mouse CD39 PE	143804	BioLegend
anti-mouse CD73 Biotin	127204	BioLegend
anti-mouse CD152 BV650	106323	BioLegend
anti-mouse CD199	128710	BioLegend
